# A Comparison of Serum Lipid Profile between Periodontitis Patients and Healthy Individuals

**Published:** 2011-04-01

**Authors:** J Hamissi, M T Shahsavarani, H Hamissi

**Affiliations:** 1 Department of Periodontics and Preventive Dentistry, Qazvin University of Medical Sciences, Qazvin, Iran; 2 Dentist, Qazvin University of Medical Sciences, Qazvin, Iran; 3 College of Dentistry, Qazvin University of Medical Sciences, Qazvin, Iran

**Keywords:** Chronic periodontitis, Hyperlipidemia, Cholesterol, HDL, LDL

Dear Editor

Recent studies conducted in our country show a prevalence of elevated lipid level or hyperlipidemia in 23.6% of adults aged 20 years and above. Since hyperlipidemia is a main risk factor for cardiovascular diseases,[1] such as atherosclerosis, cardiac ischemic disorders, and strokes, it is imperative to determine its causes. Among the main factors involved in increased levels of blood lipids are genetics, a high-fat diet and lack of physical exercise. Lately, however, the medical community has questioned whether periodontal diseases could be a risk factor for the development of hyperlipidemia. Recent studies have suggested such a relationship that most literatures support,[2][3][4][5] though two studies showed confounding results.[6][7]

In Iran, the prevalence of periodontitis in 40-69 year olds affects 70% of the general population.[7] Periodontitis is a common oral chronic infection leading to gingival inflammation, destruction of periodontal tissues, and deterioration of alveolar bones and finally loss of teeth.[8] This disease develops when dental plaques release bacteria responsible for the release of toxic substances and enzymes from sub-alveolar bacteria groups. Response of the host to the infection leads to the local production of cytokines and biological intermediates such as prostaglandins and systemic responses such as the production of antibodies in serum. Considering the extent of microbial plaques associated with this disease, its chronic nature and local and systemic immunological responses of the host, it is reasonable to assume that periodontal infections affect the overall health of a patient and could be involved in the development of systemic diseases such as hyperlipoproteinemia and hypertriglyceridemia.[9] Furthermore, several studies have found an association between periodontitis and an increased relative risk for cardiovascular disease, ischemic stroke,[10] and coronary heart disease.[11] A probable mechanism by which periodontitis might affect cardiovascular health is chronic oral inflammation that may lead to increased blood cholesterol levels. Total cholesterol and LDL in patients affected by these diseases were higher than those in control groups.[8] Considering the importance of finding the root causes of hyperlipidemia and increasing levels of hyperlipidemia in the general population, the aim of this study was to investigate the relationship between chronic periodontitis and serum lipid levels.

Our case-control study included 60 patients, 30 with chronic periodontitis (case group) and 30 healthy patients (control group) who referred to the division of periodontics, college of dentistry at Qazvin University of Medical Sciences. These two groups were paired regarding gender and age; and they were similar in weight, height, diet, health conditions, and teeth number. Periodontal states of the two groups were determined based on the presence of subgingivial and supragingival plaques and germs, the presence of at least one periodontal pocket with a depth equal to or more than 4 mm in each quadrant, and the evidence of alveolar bone destruction correlating with plaques and calculus as revealed by panoramic radiography.

The patients had no history of periodontal treatment and six months prior to the study were not afflicted with any systemic diseases, taking any medications to reduce serum lipids and were nonsmokers. Total cholesterol, triglyceride, LDL and HDL levels were measured in both groups using the enzymatic-calorimetric method or by single-point measurements by the photometry method in terms of mg/dL and the role of the diseases with the presentation of hyperlipidemic indices was determined in the patients. Data was analyzed using the SPSS software (version 13.0, Chicago, IL, USA) via the student t-test.

The two groups were matched regarding age; thus, the average age of the case group was 35.93±5.68 years and that of the control group was 34.7±5.59 years. Levels of total cholesterol, triglyceride, HDL and LDL in blood serum were measured in both groups and assuming a normal distribution curve, the average level for the two groups were calculated. For cholesterol and triglyceride, the odds ratios were calculated too.

The average cholesterol amounts were 202.8±33.49 mg/dl (case group) and 193.46±26.4 mg/dl (control group), with no significant difference (p=0.240). Also, normal and abnormal serum total cholesterol and the exposure odds ratio of the case to the control group was OR=3.76, 95% CI: 0.91-18.35, which considering the incorporation of 1, the odds ratio showed no difference from 1. The triglyceride level was 175.5±94.63 mg/dl (case group) and 149.5±54.86 (control group), with no significant difference (p=0.198). Furthermore, the odds ratio was also calculated at OR=1.52, 95% CI: 0.35-6.95, with no significant difference from 1.

When comparing HDL between the two groups, the average was 46.4±7.76 (case group) and 44.12±9.24 mg/dl (control group), showing no significant difference (p=0.305). HDL levels in all 60 patients were in the normal range. Regarding LDL, averages were reported as 126.53±31.48 mg/dl (case group) and 119.16±25.83 (control group), again the difference was not statistically significant (p=0.327). Only one patient in the case group had an LDL level above normal, while in all other 59 patients, their LDL levels were in the normal range.

As noted before, an altered lipid profile could be a risk factor for various diseases such as atherosclerosis and cardiac ischemic disease. However, in our study, levels of total cholesterol, triglyceride, LDL and HDL exhibited no correlation with periodontal disease. Perhaps these result discrepancies are due to different sample sizes. Our study was composed of 60 patients separated into two groups. In the two groups, a statistical difference of 0.17 and 0.16 appeared in the HDL and LDL levels, respectively. Our results indicated no significant statistical difference between the case and control groups. Most aforementioned studies have had the same problem. Another reason for the discrepancy among the results of these studies has been the severity of periodontitis in the case groups. In our study the design and the mentioned factors were also a problem. Another flaw among the existing research has been due to the case-control design in which reproducibility was difficult and determining the sequence in which periodontal disease and hyperlipidemia are exhibited. Therefore, even in cases that have lead to a significant result, determining if hyperlipidemia has been a risk factor or outcome is difficult and it seems that to achieve suitable results, different case designs such as cohort studies with a larger sample size may lead to more reliable results.
